# The Case of an Elderly Male Patient with Unknown Primary Mucinous Adenocarcinoma within Presacral Teratoma (Teratoma with Malignant Transformation)

**DOI:** 10.1155/2015/170479

**Published:** 2015-03-22

**Authors:** Ozgur Tanriverdi, Ayca Ersen, Suna Cokmert, Emine Koca, Naki Bulut, Suha Gul, Nevin Yilmaz

**Affiliations:** ^1^Department of Medical Oncology, Sitki Kocman University Faculty of Medicine, 48000 Mugla, Turkey; ^2^Department of Pathology, Dokuz Eylul University Faculty of Medicine, 35010 Izmir, Turkey; ^3^Department of Medical Oncology, Kent Hospital, 35010 Izmir, Turkey; ^4^Department of Internal Medicine, Sitki Kocman University Faculty of Medicine, 48000 Mugla, Turkey; ^5^Department of General Surgery, Sitki Kocman University Education and Research Hospital, 48000 Mugla, Turkey; ^6^Department of Radiodiagnostics, Sitki Kocman University Education and Research Hospital, 48000 Mugla, Turkey; ^7^Department of Internal Medicine and Gastroenterohepatology, Sitki Kocman University Faculty of Medicine, 48000 Mugla, Turkey

## Abstract

Teratomas are rarely seen in adults, and presacral region is an area where they rarely settle in. Similarly, only about 1% of teratomas show malignant transformation. Malignant transformation is often associated with the area where teratoma settles in. Malignant transformation of mediastinal teratomas is more frequent than the ones located in retroperitoneal area and gonad. They most commonly show rhabdomyosarcoma, primitive neuroectodermal tumor, enteric adenocarcinoma, and leukemia transformation. In teratomas showing malignant transformation, the clinical course is aggressive; and survival of patients with metastatic disease is very low. The primary treatment of teratomas with malignant transformations is surgical. Effect of radiotherapy and chemotherapy is not clear in patients, to whom surgical operation cannot be applied, or those who are with residual tumor, even if surgical operation can be applied to them, or those who are at metastatic stage. In this paper, we presented a 76-year-old male patient due to the histologic diagnosis of mucinous adenocarcinoma within teratoma, in whom approximately 7 cm presacral mass was found during the radiographic examination made by the reason of low back pain and pelvic pain.

## 1. Introduction

Tumors settled in presacral region are highly rare [1–3]. Such tumors may have developed congenitally or by acquisition. Additionally, the frequency of tumors settled in presacral area was reported to be 1/10000. Only 10% of such tumors are seen in adults, which develop usually in newborns or infants. In addition, 80% of adults with presacral tumor are women. These tumors are usually benign, and only 1-2% of them shows malignant character [1–4].

Teratomas are the tumors most commonly seen in presacral area in infants and newborns. The rate of teratoma incidence in infants and newborns was 1/35,000–40,000, with the dominance of male gender, while the ratio was determined to be 1/40,000–63,000 in adults, with a higher incidence in women [1–3]. In adults, malignant transformation rate of primary teratomas settled in presacral region was about 1%. Adult teratomas may be transformed into squamous cell carcinoma, adenocarcinoma, sarcoma, and other malignancies [4–9].

While there were numerous reports on malignant transforming, including mucinous, adenocarcinoma, or squamous, from teratomas of ovary and testicle, no case of unknown primary mucinous adenocarcinoma within presacral teratoma in an adult male was found in English literature. In this paper, we presented the case of mucinous carcinoma that developed on the basis of presacral teratoma, which we diagnosed in a 76-year-old male patient, for the purpose of emphasizing the fact that both the settlement area and the type of malignant transformation were rare.

## 2. Case

The 76-year-old male patient presented to the outpatient clinic of general surgery, with the complaints of increasing and intensifying low back pain and pelvic pain, as well as constipation, suffered for the last two months. The history obtainedfrom the patient enabled us to ascertain that he had no different symptom related to other systems, especially weight loss, rectal bleeding, and abdominal pain; and he had no systemic disease history. No lymphadenopathy, organomegaly, abdominal mass symptom, skin lesion, abnormal pulmonary, or cardiovascular finding was detected during his physical examination. The patient's blood biochemistry and complete blood count were normal. There was no peculiarity in the rectal touch, except for presacral sensitivity and swelling detected during the examination.

**Figure 1:**
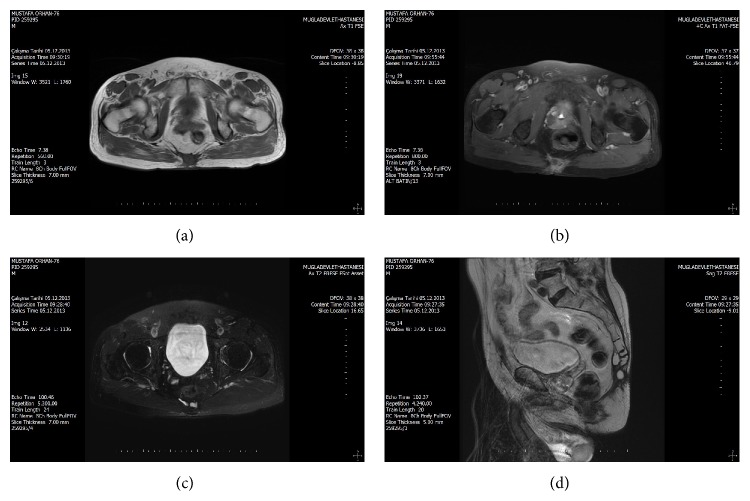
Magnetic resonance imaging. (a)–(d) A mass lesion was observed, which was separated with thin septations in the dimension of approximately 7 × 5.5 cm at* os coccyx* level, a part of which shines both in T1 and in T2, a part of which gives high signals in T1, and which had intense involvement of peripheral and septal heterogeneous contrast from postcontrast study.

**Figure 2:**
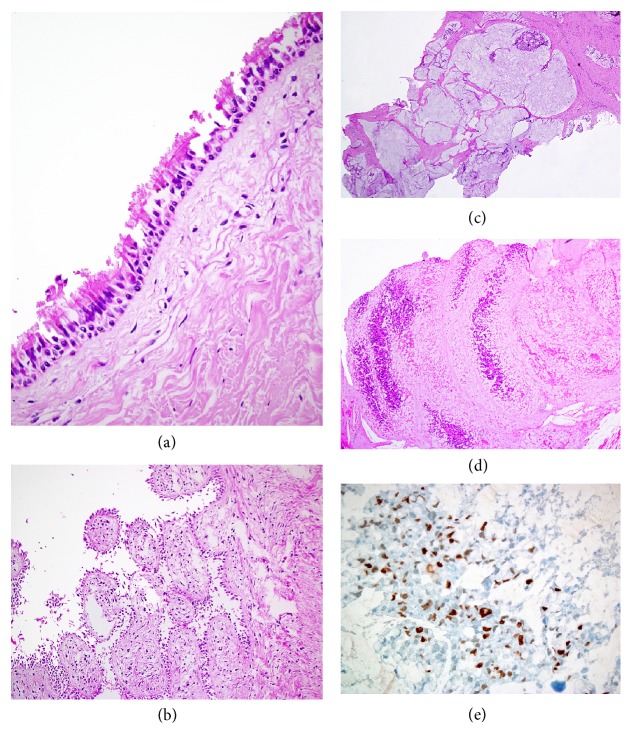
(a) The lining of some cysts was composed of ciliated columnar. (b) There were papillary structures with hyalinized stroma and ciliated columnar epithelium. (c) In some areas, there was a multiloculated cystic appearance of the tumor, most of which were filled with mucinous matrix. (d) Inside the mucin pools, there were atypical and mitotically active cells, with increased nuclear/cytoplasmic ratio; (e) the MIB-1 staining was high in these cells which were considered as malignant.

In the pelvic magnetic resonance imaging, a mass lesion was observed, which was separated with thin septations in the dimension of approximately 7 × 5.5 cm at* os coccyx* level, a part of which shines both in T1 and in T2, a part of which gives high signals in T1, and which had intense involvement of peripheral and septal heterogeneous contrast from postcontrast study (Figure 1). No other findings suggestive of malignancy were observed in his thorax and abdominal computed tomography. Total excision of the presacral mass was made (Figure 2). The histopathological diagnosis of the patient, in whom no postoperative complication was observed, was reported as teratoma containing mucinous adenocarcinoma.

Lymphadenopathy and organomegaly were not detected in the patient, who applied to the oncology outpatient department with his pathology result. His blood biochemistry and complete blood count were normal. The patient's alpha-fetoprotein, human chorionic gonadotropin hormone, cancer antigen (CA) 19-9, CA 125, carcinoembriogenic antigen, and prostate specific antigen levels were within normal limits. Any finding related to malignancy was observed in the esophagus, stomach, and the colorectal during the endoscopic examination of the upper and lower gastrointestinal systems. The testicular ultrasonography was normal. No primary tumor or metastatic lesion was detected in terms of mucinous adenocarcinoma, by the performed positron-emission tomography/computerized tomography. Adjuvant systemic chemotherapy was recommended to the patients with negative surgical margins. The therapy was commenced with the biweekly application of the regimen containing irinotecan 185 mg/m^2^/day, d1, 400 mg/m^2^/day, d1 and 2800 mg/m^2^, d1-d2 infusional fluorouracil. The patient is still under treatment and the metastasis is being monitored for 10 months.

### 2.1. Histopathological Examination

On microscopically examination the mass was located in the subcutaneous area and it had an appearance of a multiloculated cyst with some areas being more solid. The cystic areas were lined by either ciliated columnar epithelium with serous nature or mucinous epithelium. There were some large mucin pools. Some of these mucin pools contained some atypical cell groups which were considered malignant based on the morphological and immunohistochemical staining (diffuse MIB-1 staining and p53 positivity). The tumor was reported as a sacrococcygeal subcutaneous teratoma with malignant mucinous adenocarcinoma (Figure 2). Status of isochromosome 12 could not be tested due to technical problems.

## 3. Discussion

In this paper, we discussed our 76-year-old male patient diagnosed with mucinous carcinoma developing on teratoma, as a result of total presacral mass excision.

Teratomas are tumors containing different types of cells developing from multiple germinal layers. Most of teratomas are seen in testicles and ovaries of adolescents [4, 7, 9]. However, it may also develop in sacrococcygeal, presacral, retroperitoneal, postanal or mediastinal, and pineal gland, which are also known as midline structures. Different hypotheses were proposed, in regard to the development of teratomas. “Germ cell theory” is the most widely accepted one proposing that primitive totipotential cells act as a “wandering germ cell,” arisen from cells left behind during migration of embryonic germ cells from yolk sac to the gonads [9].

Primary presacral teratomas are highly rare in adults. Its malignant transformation rate is about 1% [9]. In the literature, we did not find any case of presacral teratoma that showed malignant transformation, in older adults. Our case gains importance due to this rare situation.

Presacral teratomas are often asymptomatic in adults, and they are usually detected during clinical or radiological examination. Although 90% of sacrococcygeal teratomas are externally visible in neonatal period, most of the teratomas in this region are presented as pelvic mass in adults [1–4, 10]. The most frequent symptom was reported to be pain. The pain is severe increasing with movement, in the back, lower back, and pelvic area. However, the other symptoms are mild and they are considered as nonspecific [1–4, 10]. Symptoms related to compression of organs located around presacral mass may occur [8, 11]. Constipation, sensation of incomplete evacuation, narrowed stools, or incontinence is among the possible symptoms. Frequent urine, dysmenorrhea, vomiting, nausea, edema, and paresthesia in the lower extremities are the other possible symptoms [12]. Our patient applied to the general surgery polyclinic, with the complaints of low back pain, pelvic pain, and constipation.

Presacral cystic teratomas should be distinguished from ependymomas in men and from ovarian cancer in women. However, in both sexes, meningocele, rectal duplication, tail-gut cyst, neurogenic tumors, osseous lesions, renal cysts, the Mullerian ducts cysts, and epidermal cysts are the reasons for benign disorders that they may encounter. Besides these, also lymphangioma, sarcoma, mucinous adenocarcinoma, cystic mesothelioma, metastatic lymphadenopathy, ovaries or uterus metastatic tumors listed as Wilms tumor, and soft tissue malignancies should be distinguished [4, 9, 13–17]. In addition, sometimes perirectal abscess, granulomata, fistulas, and sometimes tuberculosis cases, and so forth, may arise [13–17]. And extraneural myxopapillary ependymoma thought to be derived from embryological residues that can be seen in sacrococcygeal area should be considered during the definitive diagnosis, as well. Particularly teratomas in mucinous content may create a diagnosis difficulty, with myxopapillary ependymoma [4–8, 13, 15, 17].

Our patient was diagnosed with adenocarcinoma on the teratoma surface, during the histopathological examination. Malignant tumor finding was not observed in the testicle, colon, stomach, and pancreas during the radiological and endoscopic examinations intended for primer. Tumor markers were not guiding. Our patients was evaluated to be a patient with unknown primary cancer.

No case of mucinous adenocarcinoma transformation in teratoma was found in the literature. However, two cases with retroperitoneal adenocarcinoma transformation have been reported [9, 16]. Metastasis frequency is high, and clinical course is highly aggressive. Previous studies reported that prognosis is poor in metastatic patients and that the average survival time is limited to 28 months [10, 14].

Altman et al. [18] have classified sacrococcygeal teratomas in 4 categories. In Type 1 patients, predominant external tumor accompanied with minimal presacral component is detected, whilst Type 2 patients are presented externally—however, their intrapelvic extension is explicit. In Type 3 patients, externality is still explicit but pelvic mass extending into the abdomen is predominant. Type 3 is the category most frequently seen in adults. On the other hand, Type 4 patients are presacral masses without external presentation [18]. Our patient was evaluated to be a Type 4 patient.

Histological features of teratomas showing malignant transformation have been very well defined. They are considered to be somatic components of histological features of a tumor with nonseminomatous germ cell, which cannot be distinguished from a somatic malignancy [13–17]. Malignant transformation often includes rhabdomyosarcoma, primitive neuroectodermal tumor, enteric adenocarcinoma, and leukemia. Malignant transformations may occur in any primary area, where mature or immature teratomas develop. On the other hand, mediastinal teratomas and other tumors with nonseminomatous germ cell are more likely to show malignant transformation, compared to gonadal or retroperitoneal primary tumors [9]. Although some chromosomal abnormalities such as isochromosome 12p are associated with malignant transformation and carcinogenesis has yet to be clarified [9, 17, 19, 20]. Similarly, although surgical treatment of operable patients by means of total excision is considered to be a standard, systemic chemotherapy of such patients is still complex. It is because they are resistant to standard chemotherapy [14]. However, there are a limited number of studies and case presentations showing that a significant ratio of response and survival advantages can be obtained in a group of patients through a chemotherapy regimen to be selected depending on the cell, into which the transformation will occur [9, 14]. It has been reported that cisplatin-based chemotherapy regimens are likely not to be effective enough but fluorouracil-based regimens may produce good results especially in adenocarcinoma transformation arising from retroperitoneal or presacral teratomas [20].

In conclusion, it should be remembered that teratomas can be seen in presacral spaces in adults and may develop malignant transformation even though rarely; and histopathological examination should be carried out in detail, based on this thought. No standard systemic treatment intended for teratomas showing malignant transformation is clear yet; and therefore, sharing such rare cases may create an important database.
